# Influence of Saline Buffers over the Stability of High-Annealed Gold Nanoparticles Formed on Coverslips for Biological and Chemosensing Applications

**DOI:** 10.3390/bioengineering7030068

**Published:** 2020-07-03

**Authors:** Lan Zhou, Rodica Elena Ionescu

**Affiliations:** Laboratory Light, nanomaterials and nanotechnologies - L2n, CNRS ERL 7004, University of Technology of Troyes, 12 rue Marie Curie, 10004 Troyes, France; lan.zhou@utt.fr

**Keywords:** stability of annealed gold nanoparticles, saline buffers, BPE sensing

## Abstract

Herein, coverslips were used as solid supports for the synthesis of gold nanoparticles (AuNPs) in three steps: (i) detergent cleaning, (ii) evaporation of 4 nm gold film and (iii) exposure at high annealing temperature (550 °C) for 3 h. Such active gold nanostructured supports were investigated for their stability performances in aqueous saline buffers for new assessments of chemical sensing. Two model buffers, namely saline-sodium phosphate-EDTA buffer (SSPE) and phosphate buffer saline (PBS), that are often used in the construction of (bio)sensors, are selected for the optical and microscopic investigations of their influence over the stability of annealed AuNPs on coverslips when using a dropping procedure under dry and wet media working conditions. A study over five weeks monitoring the evolution of the localized surface plasmon resonance (LSPR) chemosensing of 1,2-bis-(4-pyridyl)-ethene (BPE) is discussed. It is concluded that the optimal sensing configuration is based on annealed AuNPs exposed to saline buffers under wet media conditions (overnight at 4 °C) and functionalized with BPE concentrations (10^−3^–10^−11^ M) with the highest LSPR spectra after two weeks.

## 1. Introduction

Metallic nanoparticles are attractive active supports for various optical (bio) sensing applications [1,2,3]. For instance, gold nanoparticles (AuNPs) are responsible for improved sensitivity and stability of optical sensing properties [4,5,6] and imaging [7]. Thus, AuNPs represent excellent nanoplatforms in the development of various configurations of sensing chemicals and biomolecules including viruses [8,9]. In this context, the localized surface plasmon resonance (LSPR) approach is often used in (bio)sensing diagnostics by monitoring the changes in AuNPs size, shape, composition, inter-particle distance, dielectric constant (refractive index) of the surrounding medium [8,10] and the linear and/or non-linear optical properties [11]. However, before home-made AuNPs can be successfully used in clinical studies, several issues should be addressed in terms of reproducibility, reliability and stability.

In general, the most common synthesis method of AuNPs is to chemically reduce the metal salt in a solution containing a stabilizer, thereby limiting the nanoparticle’s grain growth, controlling its shape and improving its stability [12]. Reducing agents, such as hydrogen, hydrazine, alcohol, carbon monoxide, Li AlH_4_, NaBH_4_ or R_4_N^+^ (Et3BH^−^), have been used to control the nano-size range [13]. For instance, colloidal AuNPs were synthesized by reacting chloroauric acid solution (HAuCl4) with sodium citrate solution as the reducing and stabilizer agents [14,15,16]. Furthermore, on the basis of the particle diameter and the dielectric function of AuNPs, resonance elastic light scattering (RELS) was used to monitor the ligand-exchange processes of the interactions of AuNPs with homocysteine, cysteine and glutathione biomarkers. 

Although chemical reduction is convenient for synthesizing nanoparticles, it also has certain disadvantages. In addition to simplicity, this method requires extreme conditions such as high temperature and pressure, and it takes a long time to complete the reaction. The most important drawback is the nature of the reactants used in the reaction system. They are usually toxic chemicals that present potential hazard to the environment and health [17,18]. 

On the other hand, the aggregation of colloidal AuNPs has been used for colorimetric [19], electrochemical [20] and atomic force microscopy [21] investigations. In such applications, the choice of buffer composition is crucial for analytical performances of the sensing scheme. For example, SSPE and PBS saline buffers have been widely for dissolving, diluting or storing active biomolecules [22,23,24]. In other cases, buffers were used to study the effect of electrocatalytic performances of modified electrodes for electrochemical biosensing [25,26,27,28]. However, the effect of saline buffers on the size and dispersion stability of nanoparticles on solid supports has not yet been studied. 

The present paper reports on the influence of the aqueous buffers SSPE and PBS over the stability of high-annealed 4 nm gold film-coated coverslips using on optimized AuNPs synthesis protocol [29]. As it is stated in the experimental section, the annealed gold coverslips exhibit either pinkish colors when uniformity is formed or blue colors when aggregated after buffer exposure. SEM and atomic force microscope (AFM) imaging of AuNPs are collected and analyzed after dry and wet media working conditions. Furthermore, an example of BPE (1,2-bis-(4-pyridyl)-ethene) chemical sensing under optimal conditions is also discussed.

## 2. Materials and Methods

### 2.1. Chemicals 

Liquid detergent Decon 90 (Decon Laboratories™ Decon 90™) was provided by Fisher Scientific (Göteborg, Sweden). Sodium chloride (NaCl, purity 99.5%), sodium phosphate monobasic and dibasic (purity 99.0%) and ethylenediaminetetraacetic acid (purity 99.0%) were provided by Sigma-Aldrich (St. Louis, MO, USA), while *trans* 1, 2-bis-(4-pyridyl)-ethene (BPE) was purchased from Sigma-Aldrich (Schnelldorf, Germany). Other chemicals were of analytical grade purity. Squared glass coverslips were provided by Carl Roth GmbH +Co. KG, (Karlsruhe, Germany).

In the preparation of saline buffers and cleaning of coverslips, ultrapure distilled water (18.2 MΩ·cm) was used, here named dd-water and produced by a Millipore Milli-Q water purification system (Molsheim, France).

### 2.2. Instruments

Metal evaporation was performed with Plassys MEB 400 (Bestek, France) while a hot plate (Thermo Fisher Scientific, Waltham, MA, USA) was used for thermal annealing of gold films under clean room conditions. 

Nanostructured coverslips were characterized with a scanning electron microscope (SEM) (FEG-SU8030, Tokyo, Japan) and an atomic force microscope (AFM) (Bruker ICON, Billerica, MA, USA) with cantilever ScanAsyst-Air in silicon nitride with a tip height of 2.5–8.0 mm. A spring constant of 0.4 N/m and a reflective aluminum coating on the backside in standard ScanAsyst-Air mode were used to characterize the morphology of AuNPs. The AFM PeakForce mode was employed for the topographical characterization of annealed coverslips. 

**Figure 1:**
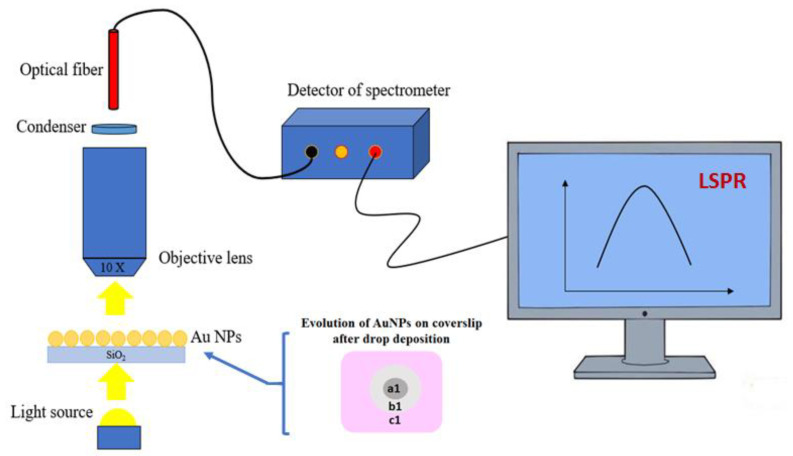
Set-up for localized surface plasmon resonance (LSPR) investigations of annealed gold nanoparticles (AuNPs) on coverslips exposed to drops of aqueous and saline buffers. Once the different drops of buffers were collected from the coverslips, three areas were observed and named **a1** (under the drop), **b1** (in the vicinity of the drop) and **c1** (far from the drop).

LSPR measurements (Figure 1) were performed with a Ø = 50 µm fiber QP50-2-UV-BX (Ocean Optics, EW Duiven, The Netherlands) coupled with an optical spectrophotometer Maya 2000 Pro and using a white exciting light source (DH-2000-BAC, Ocean Optics, EW Duiven, The Netherlands). All spectra were recorded with a 10× objective. 

### 2.3. Data Analysis

SEM images were used for estimation of the size distribution of annealed nanoparticles using the Public Domain ImageJ software developed by the National Institutes of Health based on the SEM images. 

Collected AFM images (1 µm × 1 µm) were analyzed using the Gwyddion software that adjusts the colour scale according to the maximum and minimum height per line scan. This causes regions on nanostructured glass coverslips to appear darker (i.e., lower) between nanoparticles. In addition, two parameters (R_a_ and R_rms_) were used to estimate the surface roughness of the annealed coverslips either in air for naked AuNPs or after exposure to water or aqueous buffers (SSPE and PBS). These parameters inform about the roughness of coverslips after different buffer treatments and count the distance between nanoparticles and valleys. Larger distances correspond to rougher surfaces. 

R_a_ is defined as the average roughness in µm describing the overall profile height characteristics (less sensitive to large structures and valleys), while R_rms_ is the root mean square roughness of the annealed surface averaged between the height deviations and the mean line/surface taken over the evaluation length/area.

### 2.4. Preparation of Saline Buffers

The stock 20× SSPE buffer contains 3 M sodium chloride, 0.23 M sodium phosphate dibasic and 25 mM ethylenediaminetetraacetic acid dissolved in dd-water, pH 7.4. For all experiments, 1× SSPE buffer was freshly prepared. 

PBS buffer contains 1.5 M sodium chloride, 81 mM sodium phosphate dibasic and 19 mM sodium phosphate monobasic dissolved in dd-water, pH 7.4. Five concentrations of BPE solutions (10^−3^, 10^−5^, 10^−7^, 10^−9^ and 10^−11^ M) were prepared from 97% concentrated BPE stock using dd-water as the diluting solvent.

### 2.5. Preparation of Annealed Gold Nanostructures on Coverslips 

There are three main steps for the fabrication of gold nanostructures on coverslips. (i) detergent cleaning: squared glass coverslips were degreased with an aqueous solution of Decon90 detergent (2:8 *v/v*, dd-water/detergent) in an ultrasonic distilled water bath Elmasonic S30H (Elma Schmidbauer GmbH, Singen, Germany) at 50 °C for 15 min, followed by three times ultrasonication in a dd-water bath for 5 min at 50 °C [29]. Further, each coverslip was extensively rinsed with dd-water, dried under a nitrogen stream and placed on a hot plate at 100 °C for 10 min. (ii) gold evaporation: the cleaned substrates were fixed on a circular evaporation plate, subsequently scotch-labelled at the bottom and used for coating with gold thin (4 nm) films in an evaporator (Plassys MEB 400) using a high purity 99.99% gold source (Neyco, Vanves, France) under 1 × 10^−6^ Torr pressure at 25 °C with an evaporation rate of 0.01 nm/s. (iii) annealing: the 4 nm gold-coated coverslips were thermally annealed on a hot plate at 550 °C for 3 h.

### 2.6. Effect of Buffers over the Stability of Annealed Gold on Thin Glasses 

**Figure 2:**
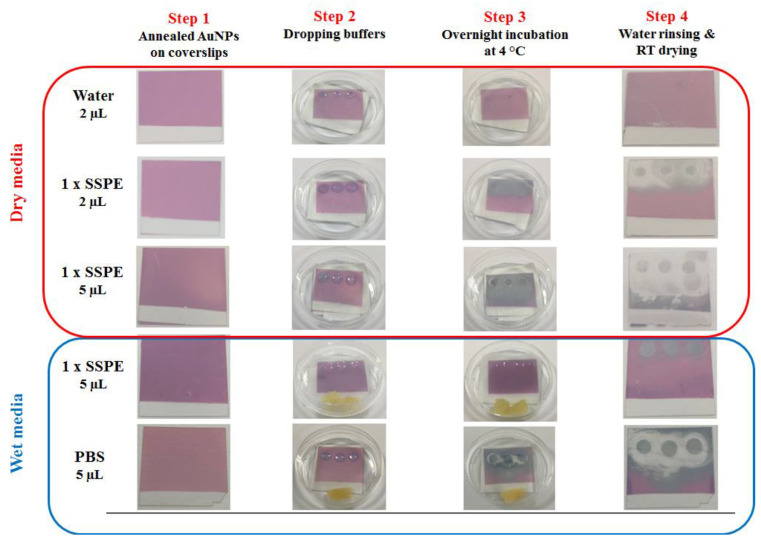
Naked-eye coverslips coated with annealed gold nanoparticles and exposed to wet and dry media for an overnight at 4 °C.

The influence of drops of water (2 µL), and 1× SSPE (2 and 5 µL) and PBS (5 µL) buffers on annealed gold nanostructures on coverslips was investigated using two approaches with a common step, an overnight exposure at 4 °C, in the presence of either (i) humid chamber petri dish—named “wet media”— or (ii) no petri dish—named “dry media” (Figure 2).

## 3. Results and Discussions

### 3.1. Characterization of Annealed Gold Coated Coverslips—Bare AuNPs 

**Figure 3:**
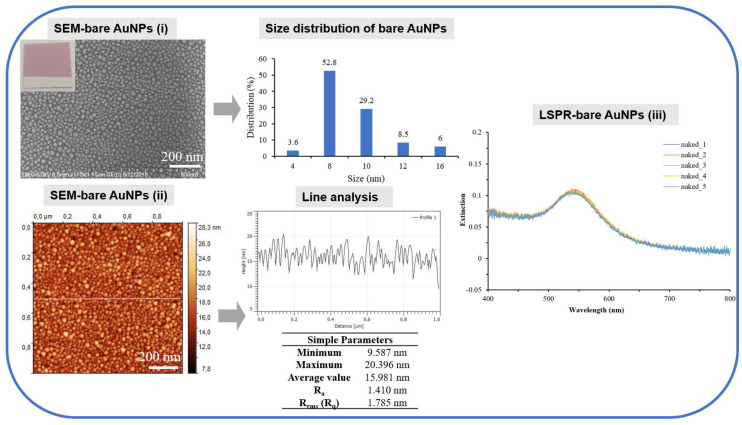
SEM (**i**), atomic force microscope (AFM) (**ii**) and LSPR (**iii**) characterizations of annealed gold nanostructured (AuNPs) coverslips. The size distribution of AuNPs (based on SEM image) and line profile analysis (based on AFM images) are also presented in the middle panel. The SEM, AFM and LSPR measurements were performed under cleanroom conditions at 21 °C.

Microscopic (SEM, AFM) and optical (LSPR) characterizations of annealed 4 nm gold-coated coverslips were performed (Figure 3). The SEM and AFM images depict a homogeneous distribution of spherical nanoparticles that are confirmed by the size distribution in the range of 4–16 nm, where a majority of AuNPs (82%) exhibit a diameter of 8–10 nm. 

The AFM image and recorded LSPR spectra also confirm the homogeneity of AuNPs from the line profile analysis with 1.410 nm R_a_ and 1.785 nm R_rms_ roughness values and from the overlapping of five LSPR spectra collected from five different areas of an annealed coverslip with the maximum plasmonic resonance at 542 nm. 

### 3.2. Influence of Water and Saline Buffers over the Stability of Annealed AuNPs on Coverslips

#### 3.2.1. Study of Water Solvent 

**Figure 4:**
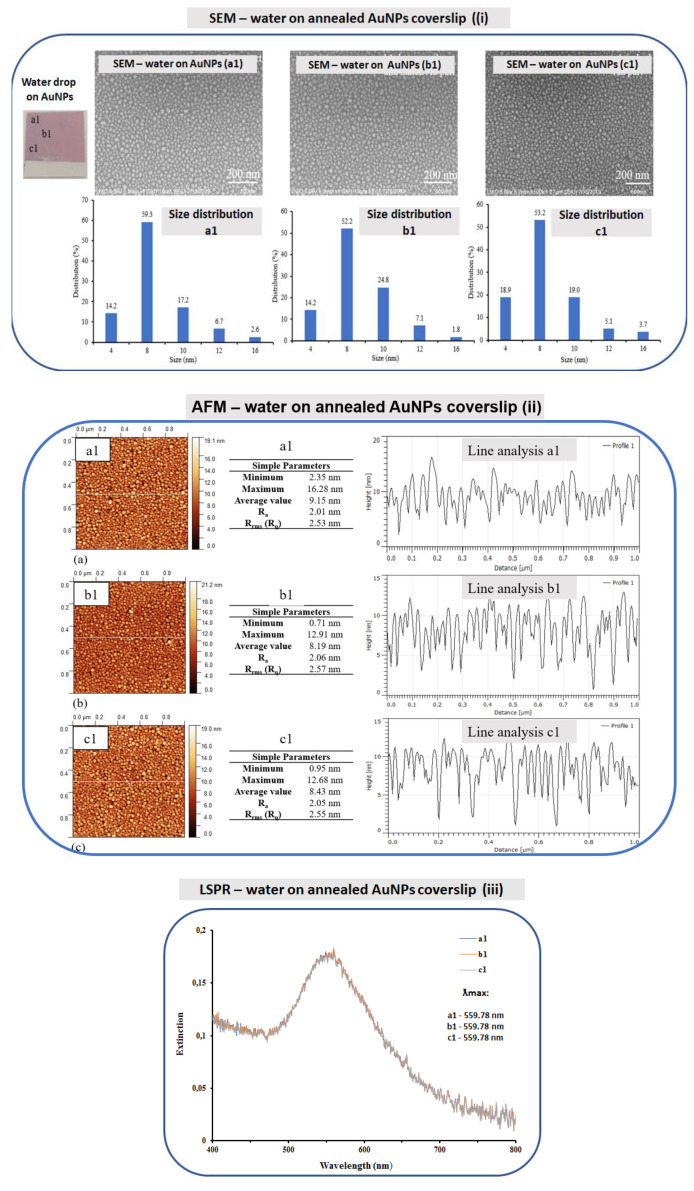
SEM (**i**), AFM (**ii**) and LSPR (**iii**) characterizations of annealed gold nanostructured (AuNPs) coverslips exposed to water drops, air dried and with three investigated areas: a1 (under the drop), b1 (in the vicinity of the drop) and c1 (far from the drop). The size distribution of AuNPs (based on SEM image) and line profile analysis (based on AFM images) were acquired for a1, b1 and c1 (dd-water, pH = 7.0). The SEM, AFM and LSPR measurements were performed under cleanroom conditions at 21 °C.

Several surface characterization techniques were employed for the nanostructured coverslips exposed to dd-water drop (2 µL) for several hours at 4 °C: SEM imaging with emphasized AuNPs size distribution, AFM imaging with the height profile analysis and LSPR collecting spectra (Figure 4). More specifically, on the coverslip, three areas were characterized: a1, just under the drop, b1, in the vicinity of the drop, and c1, far from the drop. 

It is noticed from the SEM imaging (Figure 4i) very similar morphologies of nanoparticles whatever the tested position. Thus, the nanoparticles are spherical and homogeneously distributed. This is confirmed by the AuNPs size distribution analysis, for which no significant differences were observed. The diameter of the nanoparticles is in the majority 8–10 nm and the distribution is quite narrow. The deposition of the water drop has no significant effect on the geometry of nanoparticles and does not influence their stability on the coverslip substrate. 

These observations are also confirmed by the AFM imaging (Figure 4ii) with very similar R_a_ (nm) (a1-2.01; b1-2.2.06; c1-2.05) and R_ms_ (nm) (a1-2.53; b1-2.57; c1-2.55) roughness values of the three selected areas. Concerning the LSPR spectra (Figure 4iii), a similar plasmonic peak evolution (λ_max_ at 559.78 nm) of the a1, b1 and c1 areas with optical density (OD) maximum values of 0.183 for NPs (a1-0.182; b1-0.182; c1-0.179) was recorded. 

#### 3.2.2. Study of SSPE Buffer

The SEM images in area a1 show that AuNPs form worm-like aggregates of almost ten AuNPs particles when dropped with 1× SSPE buffer under dry media (2 and 5 µL), whereas they form smaller aggregates under wet media (5 µL). 

The SEM images in area b1 show a similar morphology under dry media than this one observed under wet media in area a1. AuNPs are almost absent on the substrate for the highest buffer concentration under the dry condition. Under the wet condition, the aspect of the AuNPs is very similar to this one observed for the annealed AuNPs in Figure 2. 

**Figure 5:**
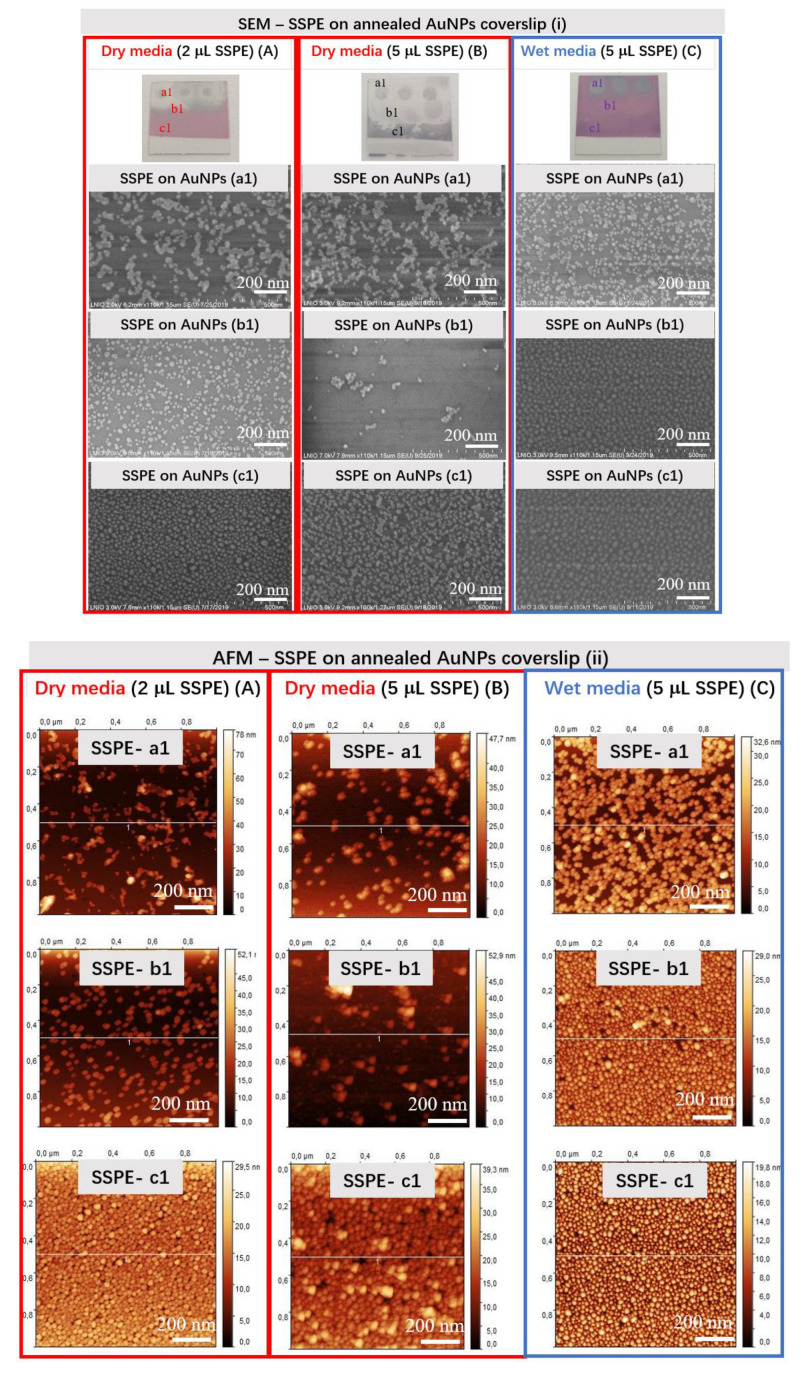

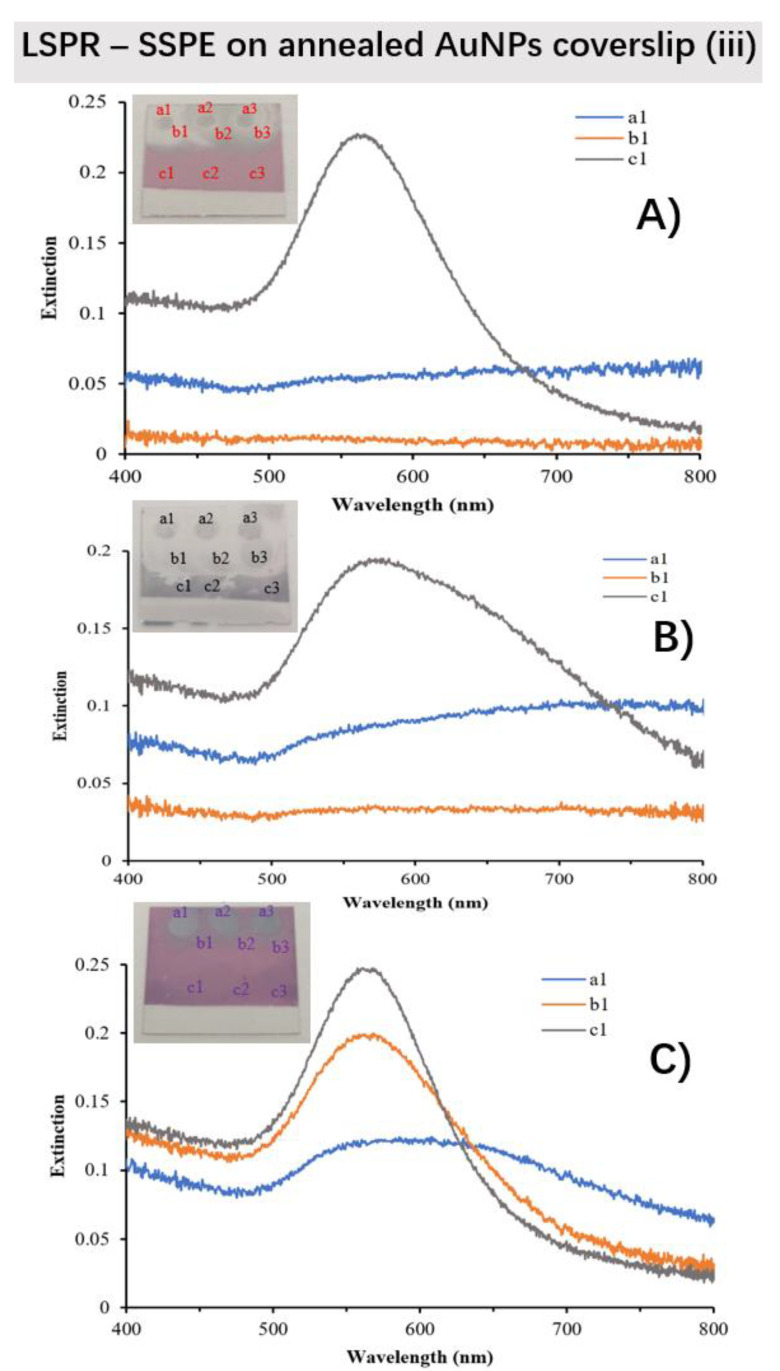
SEM (**i**), AFM (**ii**) and LSPR (**iii**) characterizations of annealed gold nanostructured (AuNPs) coverslips exposed to three drops of SSPE per coverslip with three areas investigated for each deposited/removed drop (a1, under the drop, b1, in the vicinity of the drop, and c1, far from the drop). Two approaches were tested (**A**) 2 μL SSPE drop and (**B**) 5 μL SSPE drop on coverslip for an overnight at 4 °C under dry media; (**C**) 5 μL SSPE drop on coverslip for an overnight at 4 °C under wet media (1× SSPE buffer, pH = 7.4). The SEM, AFM and LSPR measurements were performed under cleanroom conditions at 21 °C.

The SEM images (Figure 5i) in area c1 show a similar morphology for 1× SSPE buffer under dry media (A) and for the wet media (C) than was also observed for the annealed AuNPs in Figure 2. For the 5 µL buffer under dry media (B), the images evidence some aggregates. 

To conclude, the dropping of 1× SSPE buffer clearly affects the morphology and the stability of annealed AuNPs on coverslips, especially under dry media and strongly in the presence of 5 µL buffer under wet conditions. 

The size distribution of AuNPs (based on SEM image) and line profile analysis (based on AFM images) (Figure 5ii) were also acquired for the a1, b1 and c1 areas. Moreover, the variation in the R_rms_ values (data not shown), that are similar for the dry (b1—4.977 nm) and wet media (b1—3.852 nm), showed no further changes on area c1 but strongly affected area a1 for both conditions. The highest R_rms_ value is observed for the buffer content of 2 µL 1× SSPE in dry condition in area a1—7.638 nm, probably due to the faster evaporation of the drop. 

As expected, similar LSPR resonant spectra with a maximum of extinction versus wavelength were obtained for the naked AuNPs presented on the c1 area of samples under dry and wet media. For the b1 area under dry media, no plasmonic peak spectra are recorded contrary to a well-defined spectrum for the sample under wet media. Concerning the a1 zone for all tested samples, no LSPR peak was possible (Figure 5iii). 

In conclusion, the protocol of annealed AuNPs under wet media shows good particle stability on coverslips and it was used for PBS buffer study. 

#### 3.2.3. Study of PBS Buffer 

**Figure 6:**
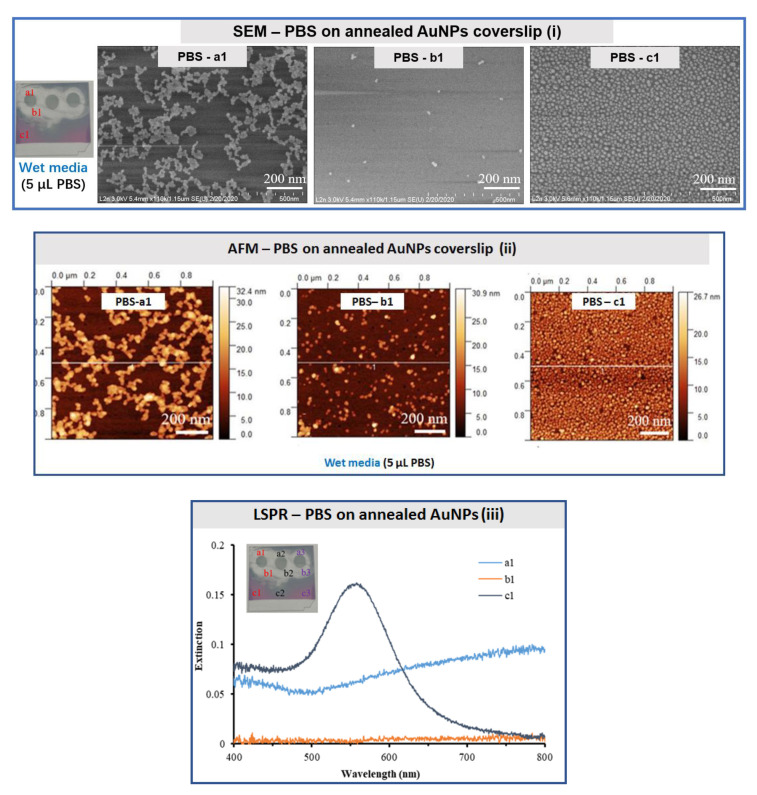
SEM (**i**), AFM (**ii**) and LSPR (**iii**) characterizations of annealed gold nanostructured (AuNPs) coverslips exposed to three drops of PBS (5 μL) per coverslip under wet media with three areas investigated for each deposited/removed drop: a1, under the drop, b1, in the vicinity of the drop, and c1, far from the drop (10× PBS buffer, pH = 7.4). The SEM, AFM and LSPR measurements were performed under cleanroom conditions at 21 °C.

Three drops of 5 μL PBS buffer were placed on annealed AuNPs and kept under wet media for overnight at 4 °C. The SEM images (Figure 6i) depict a similar behavior of agglomeration of nanoparticles in chains (a1 area) close to those images obtained in the presence of SSPE buffer (5 μL), but under dry media. Very few AuNPs are observed in the b1 area, suggesting a stronger destabilization effect of the PBS buffer over the annealed NPs. These observations are also confirmed by AFM imaging (Figure 6ii) and the corresponding line profile analysis with R_rms_ roughness values of 6.33 for a1, 1.94 for b1 and 2.91 nm for c1. 

The LSPR spectra were collected from annealed AuNPs after the deposition of three PBS drops with three tested areas (a1, b1 and c1) per drop. A resonant plasmonic peak was recorded from area c1, while no peaks were noted for areas a1 and b1 (Figure 6iii). 

### 3.3. Sensing of Chemical BPE Molecules in Aqueous Solution with SEM, AFM and LSPR Characterization

Based on the previous results on the stability of annealed AuNPs on coverslips in the presence of a water solvent, the functionalization of AuNPs in the presence of model BPE molecules in aqueous solution was investigated. 

The SEM images (data not shown) show dense, homogeneous and spherical AuNPs after treatment with BPE solutions. The NPs size distribution is distributed in a narrow range from 4 to 16 nm. For instance, the 8 nm AuNPs are the mostly formed particles on the coverslips (52.8%) and further modified by exposure to five BPE concentrations: 10^−3^ M (30.3%), 10^−5^ M (49.8%), 10^−7^ M (48.3%), 10^−9^ M (50.6%) and 10^−11^ M (46.6%). Other NPs sizes varied from 10 > 12 > 16 nm for both exposed and not exposed to BPE concentrations. No significant size differences between the naked and BPE-exposed AuNPs are observed. However, as the BPE concentration increased, the presence of gaps between the nanoparticles was observed. 

**Figure 7:**
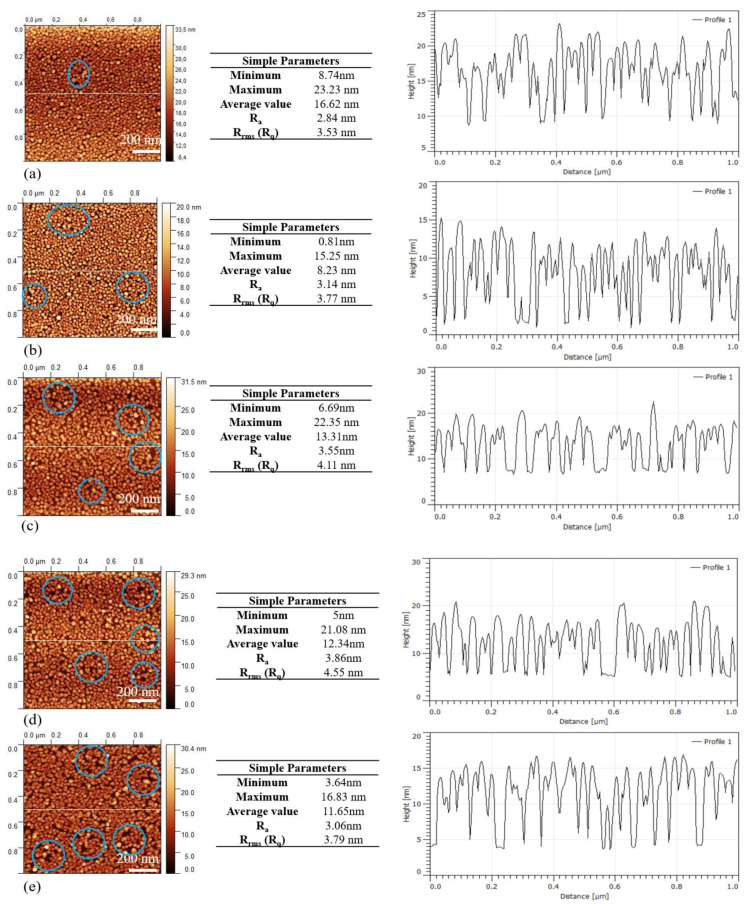
AFM images of annealed AuNPs modified by different concentrations of BPE model molecules, cross-section analysis was based on the profile line analysis. (**a**) 10^−3^ M BPE; (**b**) 10^−5^ M BPE; (**c**) 10^−7^ M BPE; (**d**) 10^−9^ M BPE; (**e**) 10^−11^ M BPE. The AFM measurements were performed under cleanroom conditions at 21 °C.

The AFM imaging of the BPE-modified AuNPs (Figure 7) confirms the homogeneous surface morphology of AuNPs on the coverslips shown with SEM images, with no large R_rms_ differences (3.53 to 4.55 nm) for the five BPE concentrations but higher than the naked AuNPs (R_rms_ = 2.55). 

**Figure 8:**
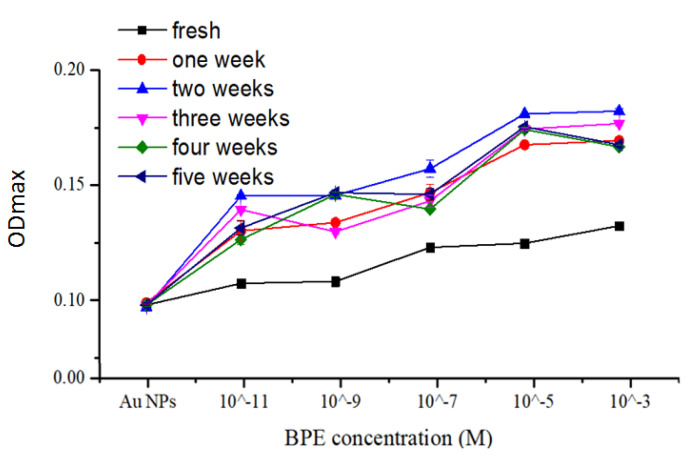
Evolution of maximum optical density (OD_max_) of annealed AuNPs functionalized with five BPE concentrations over five weeks (fresh—the day of functionalization). The coverslip was evaporated with 4 nm Au and annealed at 550 °C for 3 h.

The evolution of LSPR spectra of annealed AuNPs exposed to BPE concentrations over five weeks was investigated (Figure 8). Moreover, the values of the maximum resonant wavelength length with its corresponding maximum optical density (OD_max_) are reported in Table 1. Additionally, reproducible LSPR spectra were collected from larger coverslip areas (about 1 cm^2^).

The lower tested concentration 10^−11^ M of BPE is detectable using annealed AuNPs on coverslips. The OD_max_ values increased from the day of nanoparticle functionalization with five BPE concentrations up to 14 days. After two weeks, the OD_max_ values were decreasing but were still higher than those obtained in the day of functionalization (named Fresh). It is noticed that λ_max_ values increased as expected from 10^−11^ to 10^−3^ M BPE with the coated annealed AuNPs from the preparation day to the third week. After the third and fourth weeks, the plasmonic properties are strongly degraded.

To conclude, by comparing both plasmonic parameters (λ_max_, OD_max_), two weeks testing is recommended for an LSPR investigation of the stability of BPE functionalized AuNPs on coverslips.

## 4. Conclusions

In this work, high-annealed AuNPs were prepared on thin coverslips at a large scale, showing homogeneity and well-defined plasmonic resonant peaks. Knowing the important roles of biological buffers (for instance SSPE and PBS) in the aliquots and (bio) functionalization preparation steps, SEM/AFM/LSPR studies of nanoparticles stability in the presence of these buffers are herein reported. The influence of tiny drops of dd-water and BPE aqueous solutions for sensing on an annealed gold nanoparticles surface is also investigated. Dry and wet media working configurations were tested by dropping buffers on AuNPs. No effect in the presence of dd-water was remarked, while SSPE and PBS destabilized the annealed AuNPs under dry media. However, the AuNPs are less affected by saline SSPE and PBS buffers under 4 °C wet media. LSPR sensing of 10^−11^ M BPE was also possible on AuNPs with good plasmonic signal stability over two weeks. 

## Figures and Tables

**Table 1:** Evolution of LSPR spectra of AuNPs functionalized with different BPE concentrations over five weeks. The LSPR measurements were performed under cleanroom conditions at 21 °C.

[BPE]		Fresh Functionalization	After One Week	After Two Weeks	After Three Weeks	After Four Weeks	After Five Weeks
10^−3^ M	λ_max_ (nm)	548.210	549.904	550.792	553.910	552.660	552.660
	OD_max_	0.13240	0.16940	0.18220	0.17680	0.16668	0.16760
10^−5^ M	λ_max_ (nm)	548.566	549.814	550.260	553.110	552.660	552.660
	OD_max_	0.12480	0.16760	0.18100	0.17440	0.17420	0.17540
10^−7^ M	λ_max_ (nm)	550.706	549.636	548.566	553.110	559.780	552.660
	OD_max_	0.12300	0.14680	0.15720	0.14320	0.13960	0.14600
10^−9^ M	λ_max_ (nm)	551.864	551.062	550.440	553.288	559.780	559.780
	OD_max_	0.10820	0.13380	0.14560	0.12980	0.14600	0.14680
10^−11^ M	λ_max_ (nm)	551.864	539.290	542.412	548.654	559.870	552.660
	OD_max_	0.10740	0.13020	0.14540	0.13940	0.12640	0.13140
